# Possible sarcopenia and risk of new-onset type 2 diabetes mellitus in older adults in China: a 7-year longitudinal cohort study

**DOI:** 10.1186/s12877-023-04104-9

**Published:** 2023-07-03

**Authors:** Chun Luo, Rui-yan Liu, Guang-wu Zhang, Fei Hu, Yu-hong Jin, Bing-yang Liu

**Affiliations:** 1 Ningbo Medical Center Lihuili Hospital, Ningbo University, Ningbo, China; 2 Wenzhou Medical University Renji College, Wenzhou, China; 3 Cixi Biomedical Research Institute, Wenzhou Medical University, Cixi, China

**Keywords:** Diabetes mellitus, Possible sarcopenia, Older adults

## Abstract

**Background:**

Previous studies have shown that type 2 diabetes mellitus (T2DM) can cause sarcopenia; however, these conditions may have a bidirectional association. This study aimed to explore the longitudinal association between possible sarcopenia and new-onset T2DM.

**Methods:**

We conducted a population-based cohort study using nationally representative data from the China Health and Retirement Longitudinal Study (CHARLS). This study included participants aged ≥ 60 years who were free of diabetes during the baseline survey of CHARLS (2011 to 2012) and were followed up until 2018. Possible sarcopenia status was defined according to the Asian Working Group for Sarcopenia 2019 criteria. Cox proportional hazards regression models were used to evaluate the effect of possible sarcopenia on new-onset T2DM.

**Results:**

In total, 3,707 individuals were enrolled in this study, with a median age of 66 years; the prevalence of possible sarcopenia was 45.1%. During the 7-year follow-up, 575 cases (15.5%) of incident diabetes were identified. Participants with possible sarcopenia were more likely to have new-onset T2DM than those without possible sarcopenia (hazard ratio: 1.27, 95% confidence interval: 1.07–1.50; p = 0.006). In subgroup analysis, we found a significant association between possible sarcopenia and T2DM in individuals aged < 75 years or with a BMI < 24 kg/m². However, this association was not significant in individuals aged ≥ 75 years or with a BMI ≥ 24 kg/m².

**Conclusions:**

Possible sarcopenia is associated with an increased risk of new-onset T2DM in older adults, especially in individuals who are not overweight and aged 75 years or younger.

**Supplementary Information:**

The online version contains supplementary material available at 10.1186/s12877-023-04104-9.

## Introduction

Diabetes is a chronic metabolic disease caused by the progressive loss of β-cell mass and/or function due to various genetic and environmental factors, resulting in hyperglycemia [1]. Latest evidence shows that the number of individuals with diabetes is 537 million worldwide and 141 million in China, and this number is rapidly increasing, thus making diabetes, which is closely associated with disability and death, a major public health concern of the 21st century [2].

Sarcopenia is a skeletal muscle disorder characterized by progressive and generalized loss of muscle mass and strength, which is usually associated with age [3]. With the aging of the Chinese population, the prevalence of sarcopenia is rapidly increasing and has become a new research hotspot [3]. The diagnostic criteria for “sarcopenia” have not yet been standardized worldwide. To the best of our knowledge, all diagnostic criteria consider muscle mass, muscle strength, and physical performance, which are associated with poor health [4–7]. Access to reliable equipment to measure muscle mass in community settings remains a challenge; therefore, the Asian Working Group for Sarcopenia (AWGS) 2019 consensus introduced the concept of “possible sarcopenia” to allow the early identification of individuals at risk for sarcopenia and their timely intervention [6]. Possible sarcopenia refers to reduced muscle strength or poor physical performance that can be measured using simple and affordable methods in community screening and clinical practice [6]. This concept was developed to help better manage the risk of sarcopenia and improve the quality of life of patients.

Recent studies have shown that sarcopenia and type 2 diabetes mellitus (T2DM) have a bidirectional association [8]. T2DM can lead to the development of sarcopenia [9], which in turn can exacerbate diabetes [10], through possible mechanisms, including impaired glucose metabolism, insulin resistance, mitochondrial dysfunction, inflammation, and antioxidant stress response [8, 11]. Although several cross-sectional studies have reported that sarcopenia increases the risk of new-onset T2DM [12–14], it remains unclear whether possible sarcopenia increases the risk of new-onset T2DM. The concept of “possible sarcopenia” is relatively new, and its diagnostic criteria are not as strict as those of sarcopenia. The introduction of “possible sarcopenia” to explore its association with new-onset T2DM may help older adults prevent and manage the risk of the disease in a more timely manner.

Therefore, based on nationally representative data from the China Health and Retirement Longitudinal Study (CHARLS), we aimed to investigate the longitudinal relationship between possible sarcopenia and new-onset T2DM, as defined by the AWGS 2019 criteria, among community-dwelling older adults aged ≥ 60 years in China. This study may further improve the understanding of the relationship between possible sarcopenia and new-onset T2DM and provide a basis for better prevention of T2DM.

## Methods

### Data sources and participants

This study used data from CHARLS, which is an ongoing longitudinal survey with the first round of examinations conducted in 2011–2012 (CHARLS 2011). The participants were aged at least 45 years and were selected from 28 provinces in China using the multistage stratified probability-proportionate-to-size sampling method, representing the general middle-aged and older adult population in China. This study collected high-quality sociodemographic and health-related data through one-on-one interviews using a structured questionnaire. All participants were followed up every 2–3 years after the baseline survey. Detailed information about the study design is provided in a previous report [15].

In this study, we retrospectively analyzed data from participants who were aged at least 60 years during the baseline survey of CHARLS (CHARLS 2011) and had available information at subsequent follow-up visits until 2018. The exclusion criteria were as follows: (1) aged < 60 years at baseline, (2) having T2DM or a history of T2DM at baseline, (3) lack of data to assess T2DM at baseline, (4) lack of data to assess possible sarcopenia at baseline, and (5) lack of data to assess T2DM during follow-up.

### Assessment of possible sarcopenia

According to the AWGS 2019 consensus, possible sarcopenia is defined as low muscle strength or reduced physical performance [6].

Muscle strength was assessed by measuring grip strength. Participants were asked to squeeze a mechanical dynamometer (YuejianTM WL-1000, Nantong, China) as hard as possible, and each hand was tested twice. The maximum value of four measurements was recorded. Low muscle strength was defined as handgrip strength of < 28 kg for men and < 18 kg for women [6].

Physical performance was evaluated using the 5-time chair stand test. Participants were asked to sit down and fold their arms in front of their chest. Subsequently, they were asked to stand up and sit down 5 times in a row as fast as they could without stopping and moving their arms. The time required to complete the test was recorded by the examiner. Based on the recommendations of AWGS 2019, participants were considered to have low physical performance if they required ≥ 12 s to complete the task [6]. Participants who were unable to complete the test were also considered to have low physical performance.

### Assessment of diabetes

During the follow-up, participants with incident diabetes were identified based on the following criteria: (1) higher fasting plasma glucose (FPG) level (≥ 126 mg/dL); (2) higher random plasma glucose (RPG) level (≥ 200 mg/dL); (3) higher hemoglobin A1c (HbA1c) level (≥ 6.5%)[16]; (4) previously diagnosed with diabetes; and/or (5) currently receiving hypoglycemic therapy, including traditional Chinese medicine, modern Western medicine, or both. In CHARLS, participants were instructed to undergo an overnight fasting period prior to the collection of venous blood samples. Medically trained personnel conducted the blood collection, which included assessments for FPG and HbA1c. In cases where participants were unable to comply with the fasting requirement, blood samples were still obtained, and the glucose values were treated as RPG for analysis purposes.

The onset time of diabetes was determined using the following methods: (1) Participants reported the date or age of diagnosis in response to the question, “When was the condition first diagnosed or known by yourself?“ (2) If participants reported diabetes in one survey but lacked specific dates, the onset time was calculated as the median time between the last two surveys. (3) For participants without a reported diabetes history in the questionnaire, the date of diagnosis was identified based on the testing results from blood samples collected.

### Covariates

Statistical analyses were adjusted for baseline variables that exhibited statistical differences or were previously identified as risk factors for the onset of diabetes [16, 17]. Sociodemographic information was collected using a structured questionnaire, which included variables such as sex (male or female), age group (60–69, 70–79, or ≥ 80), residence (rural or urban), marital status (married, separated, divorced, widowed, or never married), and educational level (primary and below, secondary, or university and above). Health-related factors were assessed using a structured questionnaire and blood sample tests, which included smoking (smoker or nonsmoker), alcohol consumption (yes or no), body mass index (BMI) classification (underweight, normal weight, overweight or obese), central obesity (yes or no), hypertension (yes or no), dyslipidemia (yes or no), and FPG. Based on the Chinese criteria [18], the classification of weight status was as follows: underweight (BMI < 18.5 kg/m2), normal weight (BMI 18.5–23.9 kg/m2), and overweight or obese (BMI ≥ 24 kg/m2). Central obesity was determined by waist circumference, with a threshold of ≥ 85 cm for women and ≥ 90 cm for men [18]. Participants were considered hypertensive if they had a systolic blood pressure of ≥ 140 mmHg, had a diastolic blood pressure of ≥ 90 mmHg, received antihypertensive medication, or were diagnosed with self-reported hypertension by a physician. Participants were considered dyslipidemic if their total cholesterol level was ≥ 240 mg/dL (6.2 mmol/L), low-density lipoprotein cholesterol level was ≥ 160 mg/dL (4.1 mmol/L), triglyceride level was ≥ 200 mg/dL (2.3 mmol/L), high-density lipoprotein cholesterol level was < 40 mg/dL (1.0 mmol/L) [19], currently administered lipid-lowering medications, or were diagnosed with self-reported hyperlipidemia by a physician.

### Statistical analyses

Continuous variables were expressed as medians and interquartile ranges (IQRs), as they did not conform to a normal distribution according to the Kolmogorov–Smirnov test. Categorical variables were expressed as frequencies and percentages. Baseline characteristics of participants with and without possible sarcopenia were compared using the Mann–Whitney U test or chi-squared test, as appropriate.

The cumulative incidence of T2DM was calculated using the Kaplan–Meier method and was compared between groups using the log-rank test. Subsequently, to estimate the relationship between baseline possible sarcopenia status and incident T2DM, Cox proportional hazards models were used to calculate hazard ratios (HRs) with 95% confidence intervals (CIs).Next, potential variations in the association between possible sarcopenia and new-onset T2DM were explored across different subgroups, including those based on age (< 75 or ≥ 75) and BMI (< 24 or ≥ 24). Additionally, considering that low muscle strength and reduced physical performance are two components of the diagnosis of possible sarcopenia, their individual association with T2DM was evaluated.

In all abovementioned analyses, model 1 was adjusted for age group and sex; model 2 was additionally adjusted for BMI classification, central obesity, residence, marital status, educational level, and smoking and drinking statuses based on model 1; and model 3 was further adjusted for hypertension, dyslipidemia, and FPG based on model 2. Before data analysis, we examined the model covariates for missing values. FPG had the highest proportion of missing data (33.1%), followed by central obesity (1.7%). The other covariates had missing values ranging from 0 to 1.0%. The multiple imputation method was used to impute missing data [20]. Additionally, a sensitivity analysis was conducted using the complete case analysis method (excluding cases with missing data). Furthermore, to account for potential confounding, participants with diabetes identified based on a single blood glucose or HbA1C measurement were excluded, and a sensitivity analysis was performed.

All statistical analyses were performed using R (R Foundation for Statistical Computing, version 4.2.1). A P-value of < 0.05 was considered to indicate statistical significance.

## Results

### Study population

**Fig. 1:**
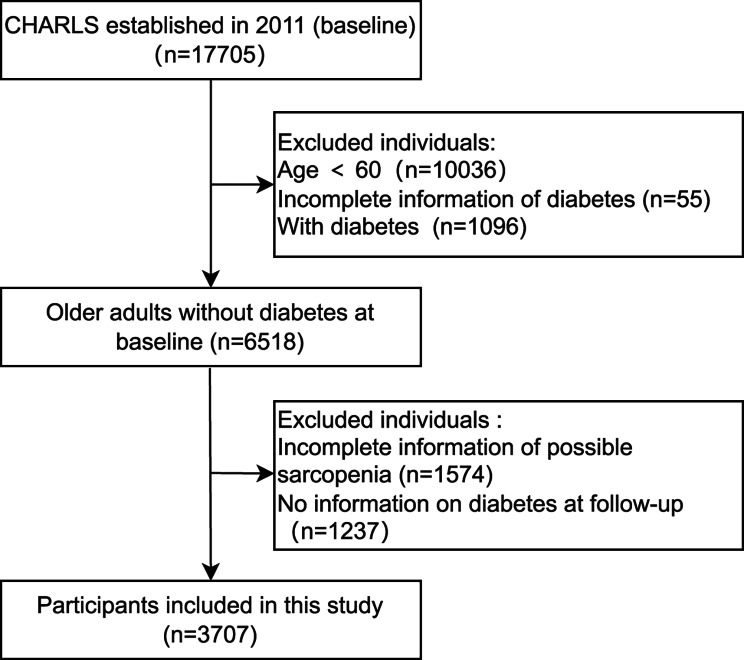
Flow diagram of the study participants

Among 17,705 participants who were interviewed during the baseline survey, we excluded participants aged < 60 years (n = 10,036), those with diabetes (n = 1,096), or those with missing data regarding diabetes (n = 55) and possible sarcopenia (n = 1,574). Moreover, we excluded 1,237 participants for whom data regarding incident diabetes were not available during the follow-up. Therefore, our final analysis included 3,707 individuals who were free of diabetes during the baseline survey and were followed up until 2018 (Fig. 1).

### Baseline characteristics of participants

The baseline characteristics of participants with and without possible sarcopenia are presented in Table 1. The median (IQR) age of the study population was 66 (62–70) years, and there were 1,805 (48.7%) men in this study. Among 3,707 older adults, the prevalence of possible sarcopenia was 45.1% (1,670/3,707). Compared with participants without possible sarcopenia, those with possible sarcopenia were older, unmarried, and women and were more likely to live in a rural area and have higher prevalence of hypertension.

**Table 1:** Baseline characteristics of participants by possible sarcopenia status

	Overall (n = 3707)	Without possible sarcopenia (n = 2037)	Possible sarcopenia (n = 1670)	*P*
Male, n (%)	1805 (48.7)	1124 (55.2)	681 (40.8)	< 0.001
Age (years)	66 [62, 70]	64 [62, 69]	68 [63, 73]	< 0.001
BMI (kg/m^2^) ^a^	22.36 [20.16, 24.90]	22.47 [20.30, 24.97]	22.20 [20.01, 24.81]	0.116
Waist circumference (cm) ^a^	84.00 [77.00, 91.20]	83.90 [77.00, 90.40]	84.00 [77.40, 92.00]	0.101
Rural, n (%)	2509 (67.7)	1343 (66.0)	1166 (69.9)	0.013
Married, n (%)	3012 (81.3)	1739 (85.4)	1273 (76.2)	< 0.001
Educational level, n (%)				< 0.001
Elementary school or below	3100 (83.6)	1616 (79.3)	1484 (88.9)	
Secondary school	562 (15.2)	384 (18.9)	178 (10.6)	
College and above	45 (1.2)	37 (1.8)	8 (0.5)	
Smoking, n (%) ^a^	1527 (41.2)	906 (44.5)	621 (37.2)	< 0.001
Drinking, n (%) ^a^	881 (23.8)	551 (27.1)	330 (19.8)	< 0.001
Hypertension, n (%) ^a^	1742 (47.1)	879 (43.2)	863 (51.7)	< 0.001
Dyslipidemia, n (%) ^a^	1571 (42.5)	858 (42.3)	713 (42.9)	0.714
FPG (md/dL) ^a^	100.98 [94.32, 108.18]	101.34 [95.04, 108.36]	100.71 [93.78, 108.18]	0.079
HbAlc (%) ^a^	5.1 [4.9, 5.4]	5.1 [4.9, 5.4]	5.1 [4.9, 5.4]	0.122
TC (md/dL) ^a^	191.37 [167.78, 214.95]	191.75 [168.94, 215.14]	190.21 [167.01, 214.56]	0.426
TG (md/dL) ^a^	100.89 [72.57, 143.37]	99.12 [72.57, 141.16]	102.66 [73.46, 144.48]	0.140
LDL-c (md/dL) ^a^	115.98 [95.10, 137.63]	117.53 [96.26, 138.02]	114.82 [93.56, 136.86]	0.066
HDL-c (md/dL) ^a^	51.03 [41.75, 61.47]	50.64 [41.75, 61.47]	51.42 [40.98, 61.47]	0.908
Handgrip strength (kg) ^a^				< 0.001
Male	36.00[31.00,41.00]	38.20[34.00,43.00]	30.50[25.00,38.00]	< 0.001
Female	24.20[20.00,28.50]	26.00[23.00,30.00]	21.35[16.50,26.00]	< 0.001
5-time chair stand test (s) ^a^	10.68 [8.75, 13.40]	9.25 [7.84, 10.43]	13.94 [12.50, 16.35]	< 0.001

Data are shown as medians (interquartile ranges) or numbers (percentages)

Abbreviations: BMI, body mass index; FPG, fasting plasma glucose; HbA1c, hemoglobin A1c; TC, total cholesterol; TG, triglyceride; HDL-c, high-density lipoprotein cholesterol; LDL-c, low-density lipoprotein cholesterol

a There were 2, 2, 5, 14, 37, 64, 66, 137, 911, 933, 933, 936, 936, and 1226 participants who missed the measurement of smoking, drinking, hypertension, dyslipidemia, BMI, waist circumference, handgrip strength, 5-time chair stand test, HbA1c, LDL-c, HDL-c, TC, TG, and FPG, respectively

### Person-years and cumulative incidences of T2DM at follow-up

**Fig. 2:**
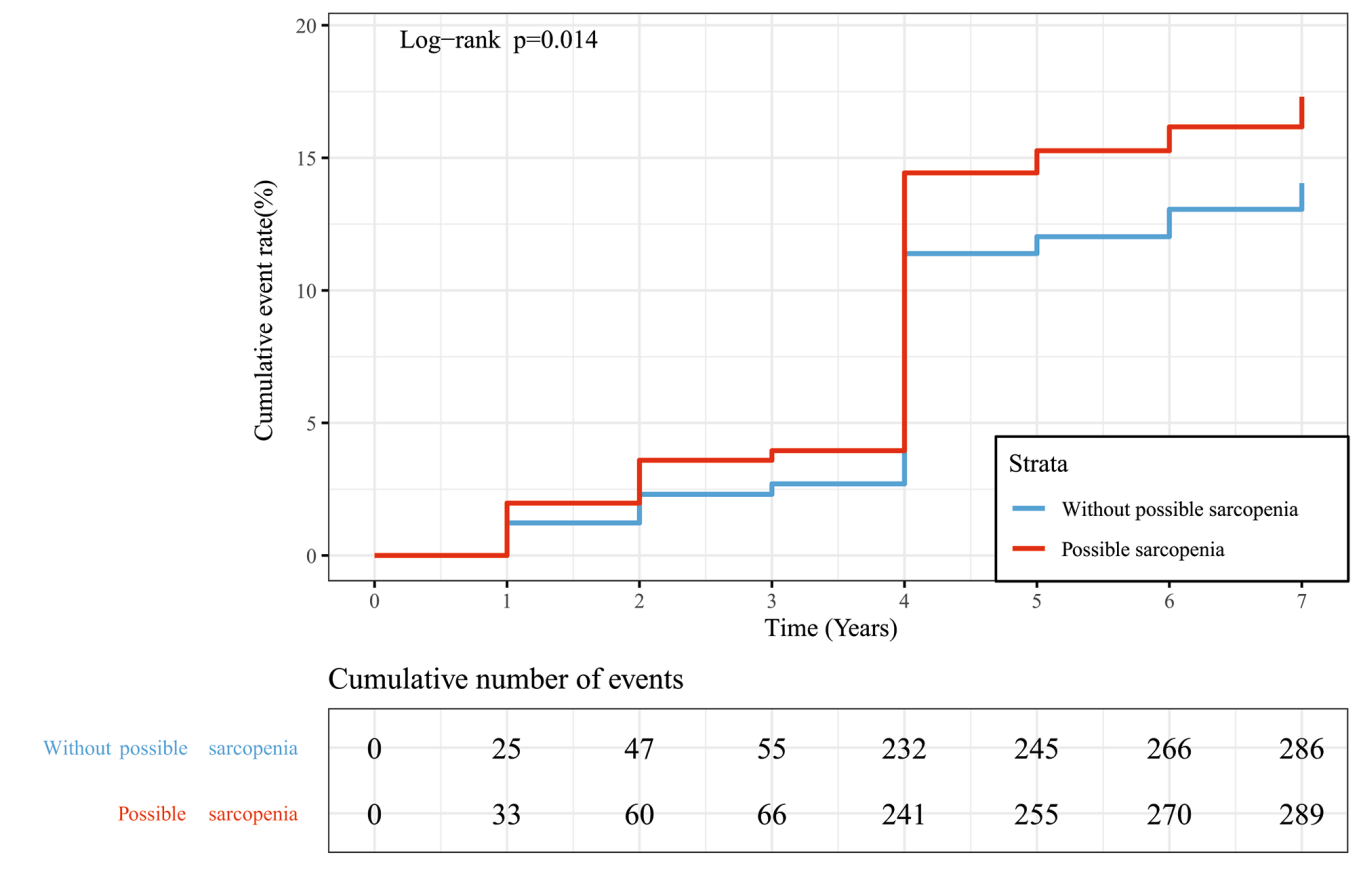
Cumulative incidence of diabetes in participants with and without possible sarcopenia at follow-up

During the 7-year follow-up, 575 cases (15.5%) of new-onset T2DM were identified. The incidence rate of T2DM was 26.85 and 21.37 per 1,000 person-years among participants with and without possible sarcopenia, respectively (Table 2). In other words, for every 1,000 older adults, possible sarcopenia was estimated to cause approximately 5 additional diabetes events annually. Data regarding the cumulative incidence of T2DM in individuals with and without possible sarcopenia from 2011 (baseline) to 2018 are shown in Fig. 2. We revealed that participants with possible sarcopenia had a higher rate of cumulative incidence of T2DM than those without possible sarcopenia (17.3% vs. 14.0%, P = 0.014).

**Table 2:** Risk of new-onset diabetes between individuals with and without possible sarcopenia

possible sarcopenia	Cases, No./total	Incidence Rate, per 1000 Person-Years		HR (95% CI)		
			unadjusted	Model 1^a^	Model 2^b^	Model 3^c^
No	286/2037	21.37	Reference	Reference	Reference	Reference
Yes	289/1670	26.85	1.26(1.07, 1.48)	1.26(1.06–1.49)	1.26(1.06–1.49)	1.27 (1.07–1.50)
p			0.006	0.008	0.008	0.006

^**a**^ Model 1 was adjusted for age group and sex

^**b**^ Model 2 was adjusted for age group, sex, body mass index classification, central obesity, residence, marital status, educational level, and smoking and drinking statuses

^**c**^ Model 3 was adjusted similar to model 2 with further adjustment for hypertension, dyslipidemia, and fasting plasma glucose

### Possible sarcopenia and the risk of new-onset T2DM

The relationship between possible sarcopenia and the risk of new-onset T2DM is demonstrated in Table 2. Compared with participants without possible sarcopenia, HR for T2DM in participants with possible sarcopenia was 1.26 (95% CI, 1.07–1.48; P = 0.006) in the unadjusted model. After adjusting for age group and sex, the HR of the possible sarcopenia group was 1.26 (95% CI, 1.06–1.49; P = 0.008). In models 2 and 3, the HRs of the possible sarcopenia group were 1.26 (95% CI, 1.06–1.49; P = 0.008) and 1.27 (95% CI, 1.07–1.50; P = 0.006), respectively.

In sensitivity analyses, we reanalyze the relationship between possible sarcopenia and new-onset T2DM using two different approaches. The complete case method (Table S1) showed an HR of 1.25 (95% CI: 1.01–1.56; P = 0.04; model 3), while excluding cases identified through a single glucose or HbA1c measurement (Table S2) resulted in an HR of 1.28 (95% CI: 1.02–1.60; P = 0.032; model 3); these associations were consistent with our main results.

### Subgroup analyses

**Fig. 3:**
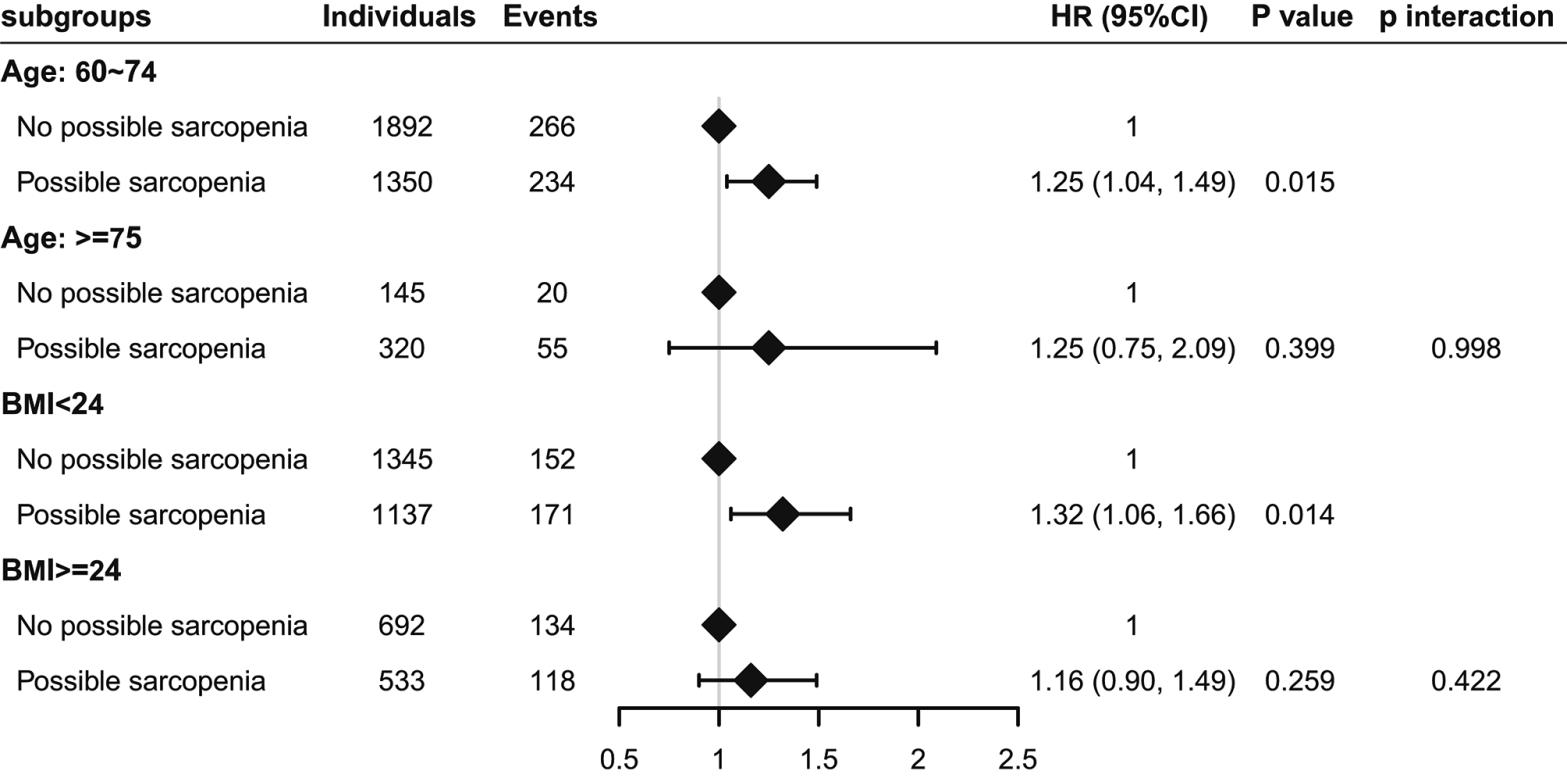
Associations between possible sarcopenia and risk of new-onset diabetes stratified by age and BMI. Forest plots display hazard ratios and 95% CIs for diabetes. Risk estimates were adjusted for age group (unadjusted in subgroup analysis stratified by age), sex, BMI classification (unadjusted in subgroup analysis stratified by BMI), central obesity, residence, marital status, educational level, smoking and alcohol consumption status, hypertension, dyslipidemia, and fasting plasma glucose. Abbreviation: HR, hazard ratio; CI, confidence interval; BMI, body mass index

In the subgroup analysis stratified by age, we found that among individuals aged < 75 years, there was a significant association between possible sarcopenia and incident T2DM, with an HR of 1.25 (95% CI: 1.04–1.49; P = 0.015). However, in individuals aged ≥ 75 years, the association was not statistically significant, with an HR of 1.25 (95% CI: 0.75–2.09; P = 0.399) (Fig. 3).

Similarly, when stratified by BMI, we observed a significant association between possible sarcopenia and incident T2DM in the group with BMI < 24, with an HR of 1.32 (95% CI: 1.06–1.66; P = 0.014; model3). However, in the group with BMI ≥ 24, the association was not statistically significant, with an HR of 1.16 (95% CI: 0.90–1.49; P = 0.259; model 3) (Fig. 3).

### Association between low muscle strength or reduced physical performance and the risk of new-onset T2DM

Participants with low physical performance did not have an increased risk of incident diabetes compared with those high physical performance (HR, 1.18; 95% CI, 1.00–1.41; P = 0.056; model 3). Similarly, we observed no significant difference in the risk of the onset of T2DM in individuals with or without low muscle strength (HR, 1.20; 95% CI, 0.96–1.51; P = 0.113; model 3) (Table S3).

## Discussion

In this study, we revealed that possible sarcopenia was associated with an increased risk of new-onset T2DM during the 7-year follow-up. This association was independent of sex, age group, BMI classification, central obesity, residence, marital status, educational level, and smoking and drinking statuses and remained significant after adjustment for fasting plasma glucose and several chronic diseases (e.g., hypertension and hyperlipidemia). In subgroup analysis, we observed a significant association between possible sarcopenia and T2DM in individuals younger than 75 years and with a BMI below 24. These results suggest that possible sarcopenia is a risk factor for T2DM. However, our study failed to determine a significant association between low muscle strength or reduced physical performance and increased risk of new-onset T2DM.

With the rapid aging of the population, possible sarcopenia has become a significant issue. The concept of possible sarcopenia has been proposed in several guidelines worldwide, including AWGS and the European Working Group on Sarcopenia in Older People. Moreover, numerous studies have investigated the epidemiology of possible sarcopenia [21–24]. Our findings revealed that the prevalence of possible sarcopenia was 45.1% in the older adult nondiabetic population in China, indicating that possible sarcopenia is a relatively common disorder, which is consistent with the findings of previous studies reporting a 46% prevalence of possible sarcopenia in community-dwelling older adults aged ≥ 60 years in China [22]. However, multiple factors, including age, genetic factors, lifestyle, nutritional status, socioeconomic status, and different research methods and criteria influence muscle strength and physical performance; therefore, the prevalence of possible sarcopenia varies across age groups, countries, and regions [9, 21]. For example, the prevalence of possible sarcopenia is 5.3% in individuals aged 40–70 years in the United Kingdom [21], approximately 25% in individuals aged ≥ 75 years in Switzerland [24], and 46.5% in individuals aged ≥ 60 years in Colombia [23].

Previous studies have shown a relationship between sarcopenia and new-onset T2DM [12–14]. These studies were cross-sectional; however, longitudinal studies can monitor changes in the same cohort and observe the evolution of the group or participants, with more convincing results. In our 7-year follow-up of 3,707 older adults in a Chinese community, we noted that the probability of developing T2DM in patients with possible sarcopenia was 17.3%, whereas that in participants without possible sarcopenia was 14%. After adjusting for as many confounders as possible (e.g., sex, age group, BMI classification, central obesity, residence, smoking and drinking statuses, fasting plasma glucose, and some chronic diseases), individuals with possible sarcopenia had an approximately 27% higher risk of developing T2DM than those without possible sarcopenia, indicating that possible sarcopenia is a risk factor for T2DM. This study was conducted using a large, nationally representative sample of Chinese individuals, indicating that the findings can be generalized to the Chinese older adult population. Our findings have significant public health implications because screening for possible sarcopenia is easy and inexpensive; thus, it can be easily extended to community health screening and routine clinical practice, which may help identify individuals at increased risk of developing T2DM who would benefit from early intervention.

Our study uncovered significant findings regarding the association between possible sarcopenia and incident T2DM across different age and BMI groups. Specifically, we observed a notable correlation between possible sarcopenia and T2DM among individuals with a BMI below 24 kg/m^2^, while this relationship was not significant among those with a BMI of 24 kg/m^2^ or higher, aligning with previous research indicating an increased risk of T2DM in non-obese individuals with sarcopenia [13]. Interestingly, we also found a clear association between possible sarcopenia and T2DM in individuals younger than 75 years, but this association was not evident among those aged ≥ 75 years. These results contradict previous cross-sectional studies conducted in a Korean population [12]. The discrepancies in findings may stem from differences in study design, population characteristics, and the adjustments made for confounding factors. Longitudinal studies like ours, which assess temporal associations, provide more robust evidence. Additionally, variations in lifestyle patterns and environmental factors could also contribute to the observed differences [25]. The complex relationship between possible sarcopenia, age, BMI, and T2DM likely involves various underlying mechanisms, such as age-related muscle changes, adipose tissue distribution, metabolic alterations, and inflammation [26]. Further research is needed to delve into these mechanisms and gain a better understanding of how age and BMI influence the association between possible sarcopenia and T2DM risk.

Possible sarcopenia is diagnosed based on physical performance and muscle strength [6]. Reduced physical performance is reported to be associated with T2DM development [27, 28]; however, the existence of a relationship between low muscle strength and T2DM development remains controversial [27, 29–31]. Some studies have suggested that low muscle strength contributes to the occurrence of T2DM [27, 29], whereas other studies have proposed that there is no association between muscle strength and the onset of T2DM [30, 31]. In the present study, we defined low muscle strength or reduced physical performance according to the AWGS 2019 criteria and followed up 3,707 older adults for 7 years. The results showed that low muscle strength or reduced physical performance alone was not statistically significantly associated with the risk of developing T2DM; however, an increasing trend was observed. This finding was inconsistent with the primary results of this study, which may be because the number of participants with low muscle strength or reduced physical performance was smaller than that of participants with possible sarcopenia; consequently, the effects of these factors on the risk of developing T2DM may be masked. Future studies should increase the sample size to explore the relationship between low muscle strength or reduced physical performance and the risk of new-onset T2DM. However, our study result was not entirely consistent with previous findings [27–31], and a relevant explanation for these inconsistent findings is the different definitions of low muscle strength and reduced physical performance [32, 33]. Our study defined low muscle strength and reduced physical performance based on the AWGS 2019 criteria; however, no previous studies have adopted this definition. Therefore, caution is needed when comparing these findings. Moreover, other possible factors, including genetics, diet, and exercise, should be considered [9, 34]. Therefore, further studies are warranted to better understand the relationship between muscle strength or physical performance and new-onset T2DM.

The mechanism by which possible sarcopenia increases the risk of incident diabetes remains unclear. The skeletal muscle is the primary site of glucose disposal, and approximately 80% of glucose is metabolized in the muscle in the postprandial state [35]. Therefore, muscle wasting may weaken the ability to maintain glucose homeostasis, particularly in the postprandial state [3]. Furthermore, sarcopenia is associated with insulin resistance [36], which is considered to be the main defect in T2DM development [37]. Furthermore, oxidative stress, inflammation, and physical inactivity may link sarcopenia to T2DM [38]. Finally, our regression analysis suggested that BMI, hypertension, and hyperlipidemia were all associated with an increased risk of T2DM. Increased BMI may lead to the development of obesity and insulin resistance, which may increase the risk of T2DM [39]. Hypertension and hyperlipidemia may contribute to the development of conditions, such as atherosclerosis and cardiovascular disease, which were also associated with T2DM development [40]. Patients with sarcopenia were more likely to have metabolic abnormalities, such as high BMI, hypertension, and hyperlipidemia, than those without sarcopenia [41]. Thus, high BMI, hypertension, and hyperlipidemia may be involved in the mechanisms by which possible sarcopenia leads to T2DM and together contribute to the development of new-onset T2DM. Future studies are warranted to elucidate the mechanisms underlying the association between possible sarcopenia and T2DM.

This study has several limitations. First, this was an observational study, and the established relationships between possible sarcopenia and incident diabetes may be biased by confounding factors. To overcome this problem, we analyzed the association by testing multiple models that included different correction factors. Additionally, we used the multiple imputation method and sensitivity analysis to minimize the offset. However, it is important to note that certain confounding factors, such as family history of diabetes, physical activity, and a fat-rich diet, were not available and could not be included in our analyses for correction. Second, there may be some bias in the diagnosis of T2DM. Medical records were not included in CHARLS, and T2DM was diagnosed using blood examinations (e.g., blood glucose and HbA1c) and structured questionnaires (e.g., self-reported physician diagnosis or current use of diabetes medication). Moreover, as blood samples were only available during baseline survey and CHARLS 2015, a higher number of new-onset T2DM cases were diagnosed in 2015 than in other years during the follow-up. To validate the reliability of T2DM diagnosis in this study, we carefully searched for studies on T2DM epidemiology in China and compared them with the present study. According to the 2010 and 2013 diabetes censuses in China, the prevalence of diabetes in individuals aged ≥ 60 years was approximately 20% [42]. However, its prevalence in 2011 in the current study was 14.4%, suggesting that the prevalence of T2DM may have been underestimated during the baseline survey. However, the rate of prevalence per 1,000 person-years during the 7-year follow-up period was approximately 23.8, which is consistent with the rate of 24.5 per 1,000 person-years reported in previous studies among urban older adults in China [43]. Although this study may have underestimated the prevalence of T2DM in older Chinese adults during baseline survey, the incidence of T2DM during follow-up was generally consistent with that of previous reports [43]; therefore, it can be considered relatively reliable for exploring the possible relationship between possible sarcopenia and new-onset T2DM. Finally, some participants with missing data on T2DM and possible sarcopenia during baseline survey were excluded from this study, which may result in a bias. Future studies are warranted to answer these important questions. Despite these limitations, this study highlights the metabolic importance of possible sarcopenia.

## Conclusion

This study provided evidence supporting a significant association between possible sarcopenia and new-onset T2DM, particularly among those who are not overweight and are younger than 75 years old. Further, our findings indicate the significance of incorporating the assessment of possible sarcopenia as a routine clinical practice in community-based health check-ups for early prevention of T2DM.

## Electronic supplementary material

Below is the link to the electronic supplementary material.

Supplementary Material 1

## Data Availability

The datasets analysed during the current study are available in the open CHARLS databases, https://charls.charlsdata.com/.
