# Supplementary material for: Possible sarcopenia and risk of new-onset type 2 diabetes mellitus in older adults in China: a 7-year longitudinal cohort study
Source: BMC Geriatr. 2023 Jul 3;23:404. doi: 10.1186/s12877-023-04104-9 (PMC10318629; doi:10.1186/s12877-023-04104-9)
Supplement: Supplementary file 1 — Supplementary Material 1 [file 12877_2023_4104_MOESM1_ESM.docx]

Supplementary Material

**Table S1. Risk of new-onset diabetes between individuals with and without possible sarcopenia when using complete cases.**

|  | **possible sarcopenia** | **Cases, n (%)** | **HR (95% CI)** | **p** |
| --- | --- | --- | --- | --- |
| **unadjusted** | No (n=2037) | 286 (14.0) | 1 |  |
|  | Yes (n=1670) | 289 (17.3) | 1.26(1.07, 1.48) | 0.006 |
| **Model 1^a^** | No (n=2037) | 286 (14.0) | 1 |  |
|  | Yes (n=1670) | 289(17.3) | 1.26(1.06–1.49) | 0.008 |
| **Model 2^b^** | No (n=1997) | 283 (14.2) | 1 |  |
|  | Yes (n=1612) | 279 (17.3) | 1.24(1.05–1.48) | 0.01 |
| **Model 3^c^** | No (n=1317) | 176 (13.4) | 1 |  |
|  | Yes (n=1107) | 183 (16.5) | 1.25(1.01-1.56) | 0.04 |

Abbreviation: HR, hazard ratio; CI, confidence interval.

**^a^** Model 1 was adjusted for age group, sex.

**^b^** Model 2 was adjusted for age group, sex, body mass index classification, central obesity, residence, marital status, educational level, smoking status and drinking status.

**^c^** Model 3 was adjusted as model 2 with further adjustment for hypertension, dyslipidemia, fasting plasma glucose.

**Table S2. Association between possible sarcopenia and incident T2DM excluding patients identified by single glucose or HbA1c measurements**

| **Possible sarcopenia** | **Cases, n (%)** | **HR (95% CI)** | | | |
| --- | --- | --- | --- | --- | --- |
|  |  | **unadjusted** | **Model 1^a^** | **Model 2^b^** | **Model 3^c^** |
| No (n=2018) | 166 (8.2) | 1 | 1 | 1 | 1 |
| Yes(n=1642) | 165 (10.0) | 1.24 (1.00, 1.50) | 1.24 (1.00, 1.56) | 1.27 (1.02, 1.59) | 1.28 (1.02, 1.60) |
| p |  | 0.052 | 0.053 | 0.036 | 0.032 |

Abbreviation: HR, hazard ratio; CI, confidence interval.

**^a^** Model 1 was adjusted for age group, sex.

**^b^** Model 2 was adjusted for age group, sex, body mass index classification, central obesity, residence, marital status, educational level, smoking status and drinking status.

**^c^** Model 3 was adjusted as model 2 with further adjustment for hypertension, dyslipidemia, and fasting plasma glucose.

**Table S3. Analysis of the risk of new-onset diabetes between the presence and absence of low physical performance, or between the presence and absence of low muscle strength**

|  | **Cases, n (%)** | **HR (95% CI)** | | | |
| --- | --- | --- | --- | --- | --- |
|  |  | **unadjusted** | **Model 1^a^** | **Model 2^b^** | **Model 3^c^** |
| **low physical performance** |  |  |  |  |  |
| No (n=2243) | 324 (14.4) | 1 | 1 | 1 | 1 |
| Yes(n=1394) | 241 (17.3) | 1.22 (1.03, 1.44) | 1.20 (1.01, 1.42) | 1.17 (0.99, 1.39) | 1.18 (1.00, 1.41) |
| p |  | 0.021 | 0.039 | 0.070 | 0.056 |
| **low muscle strength** |  |  |  |  |  |
| No (n=3084) | 469 (15.2) | 1 | 1 | 1 | 1 |
| Yes (n=557) | 94 (16.9) | 1.13(0.91, 1.41) | 1.14 (0.91, 1.43) | 1.19 (0.95, 1.51) | 1.20 (0.96, 1.51) |
| p |  | 0.276 | 0.253 | 0.121 | 0.113 |

Abbreviation: HR, hazard ratio; CI, confidence interval.

**^a^** Model 1 was adjusted for age group, sex.

**^b^** Model 2 was adjusted for age group, sex, body mass index classification, central obesity, residence, marital status, educational level, smoking status and drinking status.

**^c^** Model 3 was adjusted as model 2 with further adjustment for hypertension, dyslipidemia, and fasting plasma glucose.
